# Cost-effectiveness analysis of trastuzumab deruxtecan versus trastuzumab emtansine for HER2-positive breast cancer

**DOI:** 10.3389/fphar.2022.924126

**Published:** 2022-09-09

**Authors:** Jiangping Yang, Jiaqi Han, Yalan Zhang, Muhelisa Muhetaer, Nianyong Chen, Xi Yan

**Affiliations:** ^1^ Department of Head and Neck Oncology and Department of Radiation Oncology, Cancer Center and State Key Laboratory of Biotherapy, West China Hospital, Sichuan University, Chengdu, China; ^2^ State Key Laboratory of Biotherapy and Cancer Center, Collaborative Innovation Center for Biotherapy, West China Hospital, Sichuan University, Chengdu, China; ^3^ Department of Head, Neck and Mammary Gland Oncology, Cancer Center, West China Hospital, Sichuan University, Chengdu, China; ^4^ Clinical Research Center for Breast, West China Hospital, Sichuan University, Chengdu, China

**Keywords:** cost-effectiveness, trastuzumab deruxtecan, trastuzumab emtansine, HER2-positive breast cancer, antibody-drug conjugates, target therapy

## Abstract

**Background:** The DESTINY-Breast03 clinical trial demonstrated that trastuzumab deruxtecan (T-DXd) outperformed trastuzumab emtansine (T-DM1) in progression-free survival (PFS) in patients with human epidermal growth factor receptor 2 (HER2)-positive metastatic breast cancer (mBC). Considering the excessive cost of antibody-drug conjugates, the clinical value of T-DXd must be assessed by both its efficacy and cost. We compared the cost-effectiveness of T-DXd and T-DM1 for patients with HER2-positive mBC pretreated with anti-HER2 antibodies and a taxane from the perspectives of the United States (US) and China.

**Methods:** A comprehensive Markov model based on the DESTINY-Breast03 phase III randomized clinical trial was used to compared the cost and effectiveness of T-DXd and T-DM1 for HER2-positive mBC. Data on direct medical cost and utilities were collected from published literatures. The recorded data included the costs, quality-adjusted life-year (QALY), incremental cost-effectiveness ratio (ICER) and incremental net-health benefit (INHB). Sensitivity analysis was conducted to measure the potential uncertainty due to parameter variability. Additional subgroup cost-effectiveness analysis was performed.

**Results:** Treatment of HER2-positive mBC with T-DXd gained 0.73 QALYs compared with T-DM1 strategy. The incremental cost was $59,942 in the US, with an ICER of $ 82,112/QALY and an INHB of 0.33 QALYs, respectively. In China, the incremental cost of T-DXd versus T-DM1 was $222,680, with an ICER of $305,041/QALY and a negative INHB of -5.18 QALYs. At willingness-to-pay (WTP) threshold of $150,000/QALY in the US and $37,653/QALY in China, the probability of T-DXd as the dominant option was 77.5 and 0.1%, respectively. The unit price of T-DXd greatly influenced the results according to one-way sensitivity analysis. To meet the 50% or 90% chance of being cost-effective, the estimated cost of T-DXd would need to be less than $17.24/mg and $12.06/mg in China, respectively.

**Conclusion:** T-DXd is more cost-effective than T-DM1 for patients with HER2-positive mBC in the US, but not in China at current drug prices.

## Introduction

Breast cancer (BC) is the most frequent malignancy affecting women worldwide, with 2.26 million new cases leading to 684,996 deaths worldwide in 2020 (Sung et al., 2021). Accounting for approximately 15–20% of all invasive BC, human epidermal growth factor receptor 2 (HER2)-positive BC is closely related to aggressive tumor behavior and poor prognosis (Waks and Winer, 2019). HER2-targeted monoclonal pertuzumab and trastuzumab plus a taxane remains the first-line standard of care for HER2-positive metastatic BC (mBC) (NCCN, 2022). However, most patients experience disease progression following a response to this treatment. In relapsed or refractory disease, the antibody-drug conjugate (ADC) trastuzumab emtansine (T-DM1) was recommended as the second-line therapy according to the result of the EMILIA and TH3RESA trials (Verma et al., 2012; Krop et al., 2017). Although anti-HER2 agents have significantly improved the prognosis of advanced HER2-positive BC, resistance to these drugs develops almost inevitably, and the disease remains incurable in mBC. Therefore, further effective therapy for HER2-positive mBC, especially in later treatment, is still urgent.

Trastuzumab deruxtecan (known as DS-8201a or T-DXd) is also a HER2-targeting ADC with a humanized anti-HER2 antibody, a cleavable tetrapeptide-based linker, and a novel cytotoxic topoisomerase I inhibitor payload (Ogitani et al., 2016; Nakada et al., 2019). The US Food and Drug Administration (FDA) approved T-DXd for the treatment of patients with HER2-positive mBC who have failed two or more prior HER2-target treatments according to the results from DESTINY-Breast01 (NCT03248492) trail (FDA, 2019; Modi et al., 2020). In this phase II single arm study, T-DXd showed superior antitumor activity in HER2-positive mBC, and more than 60% of patients achieved objective response with a median PFS of 16.4 months (Modi et al., 2020). Furthermore, T-DXd was recommended as a choice for HER2-positive mBC in both the US and China by the NCCN and CSCO guidelines (CSCO, 2022; NCCN, 2022). DESTINY-Breast03 trial (NCT03529110) is the first global Phase III trial, which directly compared the efficacy and safety of T-DXd versus T-DM1 and supports the potential of T-DXd to become a new standard of care for patients who have previously been treated for HER2-positive mBC (Cortes et al., 2022). In this pivotal trail, overall response occurred in 79.7 and 34.2% of those who received T-DXd and T-DM1, respectively. At 12 months, the estimated PFS rate for T-DXd was 75.8% compared with 34.1% for T-DM1, with a HR of 0.28 (95% CI, 0.22–0.37, *p* < 0.001) (Cortes et al., 2022). The estimated OS at 12 months was 94.1% for T-DXd and 85.9% for T-DM1, respectively, with a HR of 0.55 (95% CI, 0.36–0.86, *p* = 0.007). The PFS benefits of T-DXd over T-DM1 was consistently observed across all key subgroups. In addition, the incidence rate of grade 3 or 4 adverse events (AEs) was similar between the two strategies (45.1 and 39.8%).

The T-DXd is an attractive therapeutic option that significantly decreases the risk of cancer progression and death among patients with HER2-positive mBC. However, the high price of T-DXd coupled with the relatively large patient population lead to a heavy economic burden and make it unaffordable for healthcare systems. Therefore, cost-effectiveness in healthcare is vital for decision-makers and clinicians to optimally allocate the limited medical resources. However, no relative economic analysis of treatment with T-DXd versus T-DM1 for BC has been reported previously. In this study, we compared the cost-effectiveness of T-DXd and T-DM1 for patients with HER2-positive mBC following initial treatment with trastuzumab and a taxane from the perspectives of the United States (US) and China.

## Materials and methods

### Analytical overview and model structure

A comprehensive Markov model was conducted to compare the cost and effectiveness of T-DXd and T-DM1 for patients with HER2-positive mBC (Supplementary Figure S1). We simulated a hypothetical population of patients mirror to those in the DESTINY-Breast03 trial (Supplementary Table S1) (Cortes et al., 2022). Eligible patients were included in our model and were randomly assigned to receive T-DXd (5.4 mg/kg intravenously on day 1 every 3 weeks) or T-DM1 (3.6 mg/kg intravenously on day 1 every 3 weeks) (Cortes et al., 2022). Our simulated treatment benefits were based on the PFS and OS survival curves from the clinical trial. During two initial treatments in the PFS state, patients would experience a response and continue with the therapy—either with or without grade 3 or 4 AEs until progression, unacceptable AEs or death. Upon progression or unacceptable AEs, both groups would receive subsequent treatment and best support care (BSC). As observed in the DESTINY-Breast03 trial, 29.9% (78/261) patients in the T-DXd group and 62.4% (164/263) patients in the T-DM1 group received post-study systemic anticancer treatment (Cortes et al., 2022).

**FIGURE 1:**
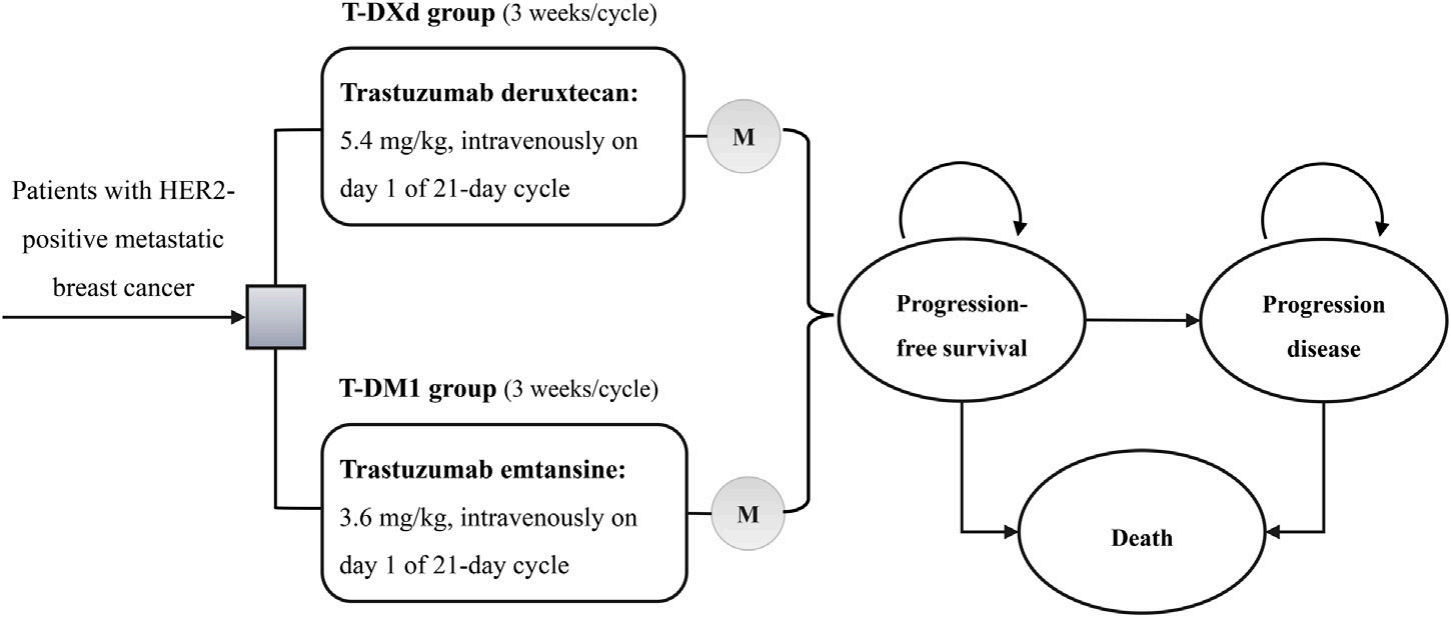
Markov model. T-DM1, trastuzumab emtansine; T-DXd, trastuzumab deruxtecan.

Three mutually exclusive health states were constructed to reflect the disease course of HER2-positive mBC: PFS, progression disease (PD), and death (Figure 1). We set the model cycle length to 21 days, which is consistent with the DESTINY-Breast03 trial. The time horizon was 10 years. The measured parameters were total costs, life-years (LYs), quality-adjusted life-years (QALYs), and incremental cost-effectiveness ratios (ICERs). We adopted half-cycle correlation and 3% annual discount rate for cost and survival estimates (Huntington et al., 2018). To estimate the cost-effectiveness of therapies, $150,000/QALY was considered the willingness-to-pay (WTP) threshold in the US (Kohn et al., 2017; Huntington et al., 2018; Wan et al., 2019; Su et al., 2021). In China, the threshold of 3× the per capita gross domestic product of China in 2021 ($37,653/QALY) were used according to the World Health Organization recommendation (Murray et al., 2000; Aguiar et al., 2018; Wu et al., 2018a; Chen et al., 2019). The model was created by TreeAge Pro (TreeAge software, Williamstown, MA), and the additional statistical analysis was carried out in R (version 4.0.3).

We considered to evaluated the incremental net-health benefit (INHB) according to the following formula: $\left(\mu_{E1}-\mu_{E0}\right)-\left(\mu_{C1}-\mu_{C0}\right)/WTP$ , where μ_Ei_ and μ_Ci_ represent the effectiveness and cost of T-DXd (i = 1) or T-DM1 (i = 0), respectively (Su et al., 2021). Also, we investigated the cost-effectiveness of subgroups based on the forest plot of the DESTINY-Breast03 trial (Cortes et al., 2022). Patients were stratified according to lines of previous therapy, visceral disease, previous pertuzumab treatment, hormone-receptor status, and stable brain metastases. Aside from PFS HRs, we assumed that the data were the same for all subgroups in the trial because of the lack of sufficient data.

### Model survival and transition estimates

The time-dependent transition probabilities among the three health states were calculated from the PFS and OS Kaplan-Meier curves of DESTINY-Breast03 trial (Cortes et al., 2022). First, we used the GetData Graph Digitizer software (version 2.26) to extract data points up to last follow-up from the curves with the method described by Hoyle et al. (Hoyle and Henley, 2011). Subsequently, the data was used to fit five common parametric survival models, including the Exponential, Weibull, Log-logistic, Lognormal, and Gompertz models (Posada and Buckley, 2004). The log-logistic distribution was determined to be the most rational function to extrapolate the survival curves because it provided the best fit based on the visual fit, the Akaike information criterion, and Bayesian’s information criterion (Supplementary Table S2 and Supplementary Figure S2). The transition probabilities between health states in each 21-day cycle were estimated according to the following formula: $1-\left\{\left[1+\lambda t^{\gamma }\right]/\left[1+\lambda {\left(t+1\right)}^{\gamma }\right]\right\}$ , where *λ* is the scale parameter, *γ* is the shape parameter and t is the current model cycle (Diaby et al., 2014). The details of the estimated model parameters are listed in Table 1.

**TABLE 1:** Key clinical and health preference data.

Parameters	T-DXd	T-DM1	References	Distribution
Log-logistic survival model				
OS of T-DXd	Scale = 0.0027363; Shape = 1.2208158		-	-
OS of T-DM1	Scale = 0.008943;Shape = 1.032004		-	-
PFS of T-DXd	Scale = 0.0075200; Shape = 1.3592687		-	-
PFS of T-DM1	Scale = 0.110280; Shape =0.957990		-	-
Grades 3–4 AEs incidence (%)				
Neutropenia	49 (19.1)	8 (3.1)	Cortes et al., (2022)	Beta
Leukopenia	17 (6.6)	1 (0.4)	Cortes et al., (2022)	Beta
Anemia	15 (5.8)	11 (4.2)	Cortes et al., (2022)	Beta
Thrombocytopenia	18 (7.0)	65 (24.9)	Cortes et al., (2022)	Beta
Nausea	17 (6.6)	1 (0.4)	Cortes et al., (2022)	Beta
Fatigue	13 (5.1)	2 (0.8)	Cortes et al., (2022)	Beta
Proportion of receiving post-study anticancer treatment (%)				
Systemic therapy	78 (29.9)	164 (62.4)	Cortes et al., (2022)	Beta
Radiation	10 (3.8)	25 (9.5)	Cortes et al., (2022)	Beta
Surgery	2 (0.8)	10 (3.8)	Cortes et al., (2022)	Beta
Rate of treatment discontinuation due to AE (%)	35 (13.6)	19 (7.3)	Cortes et al., (2022)	Beta
Utility				
PFS	0.85 (0.68–1)		Wu and Ma, (2020)	Beta
PD	0.52 (0.42–0.62)		Wu and Ma, (2020)	Beta
Discount rate (%)	3 (0–8)		Huntington et al., (2018)	Beta

AE, adverse event; OS, overall survival; PD, progression disease; PFS, progression-free survival; T-DM1, trastuzumab emtansine; T-DXd, trastuzumab deruxtecan.

### Cost and utility

The direct medical costs considered were as follows: drug acquisition costs, therapy administration, management of serious AEs, follow-up, BSC, subsequent treatment, and end-of-life care (Table 2) (Sorensen et al., 2012; Deshmukh et al., 2017; Ding et al., 2017; Wu et al., 2018b; Wu and Ma, 2020; US Department of Health and Human Services, 2022; Yaozh, 2022). The sales price of each drug in the US was calculated according to the 2022 Average Sales Price (ASP) Drug Pricing obtained from the Centers for Medicare and Medicaid Services (Centers for Medicare and Medicaid Services, 2022). The price of T-DXd in Hong Kong was used because it was not yet listed in Chinese mainland (DrugsHK, 2022). To calculate the medication doses of the drugs, a typical patient weighed and surface area of 70 kg and 1.79 m^2^ in the US, and 59 kg and 1.61 m^2^ in China was assumed for analysis (Kohn et al., 2017; The State Council Information Office, 2020). In additional, all costs in China were converted into US dollars at the exchange rate of April, 2022 (1 US dollar = 6.3509 Chinese yuan renminbi) (THE PEOPLE’S BANK OF CHINA, 2022).

**TABLE 2:** Cost estimates.

Parameters	United States ($)		China ($)		Distribution
	Mean	Range	Mean	Range	
PFS cost ($)					
T-DXd	9,305 Centers for Medicare and Medicaid Services, (2022)	7,228–10,842	10,983 DrugsHK, (2022)	8,786–13,180	Gamma
T-DM1	8,603 Centers for Medicare and Medicaid Services, (2022)	6,882–10,324	3,077 Yaozh, (2022)	2,462–3,694	Gamma
Drug administration per unit	292 Wu et al. (2018b)	234–350	18 Wu et al. (2018b)	14–22	Gamma
Routine follow-up per time	1,139 Sorensen et al. (2012)	911–1,367	166 Ding et al. (2017)	133–199	Gamma
Cost of BSC per cycle	3,230 Wu and Ma, (2020)	2,395–4,038	807 Ding et al. (2017)	646–968	Gamma
Cost of managing AEs (grades 3–4) per event					
Neutropenia/Leukopenia	17,181 Wong et al. (2018)	16,110–18,429	412 Rashid et al. (2016)	330–494	Gamma
Anemia	20,260 Wong et al. (2018)	19,295–21,378	508 Wu et al. (2018b)	406–610	Gamma
Thrombocytopenia	22,698 Wong et al. (2018)	20,289–25,377	3,395 Wu et al. (2018b)	2,716–4,074	Gamma
Nausea	19,134 Wong et al. (2018)	16,187–23,595	323 Chen et al. (2021)	258–388	Gamma
Fatigue	6,908 Mistry et al. (2018)	5,526–8,290	110 Wu et al. (2018b)	88–132	Gamma
PD cost ($)					
Systemic treatment in T-DXd	2,530 Cortes et al. (2022)	2,024–3,036	1,157 Cortes et al. (2022)	926–1,388	Gamma
Systemic treatment in T-DM1	5,640 Cortes et al. (2022)	4,512–6,768	4,022 Cortes et al. (2022)	3,218–4,826	Gamma
Radiation	7,814 Deshmukh et al. (2017); US Department of Health and Human Services, (2022)	3,907–15,628	6,298*	5,038–7,558	Gamma
Surgery	2,580 Deshmukh et al. (2017); US Department of Health and Human Services, (2022)	1,259–3,778	2,362*	1,890–2,834	Gamma
End-of-life care per patient once	9,032 Sorensen et al. (2012)	7,226–10,838	1,893 Wu et al. (2018b)	1,564–2,346	Gamma
Body weight (kg)	70 Kohn et al. (2017)	56–84	59 The State Council Information Office, (2020)	47–71	Gamma
Body surface area (meters^2^)	1.79 Kohn et al. (2017)	1.78–1.80	1.61 The State Council Information Office, (2020)	1.60–1.62	Gamma

AE, adverse event; BSC, best support care; OS, overall survival; PD, progression disease; PFS, progression-free survival; T-DM1, trastuzumab emtansine; T-DXd, trastuzumab deruxtecan. * The costs of radiation and surgery were estimated based on the price of West China Hospital Sichuan University, 2022.

Grade 3–4 AEs that occurred in over 5% of patients and had significantly different rates between treatments were included (Table 1). Under these conditions, the costs of managing neutropenia, leukopenia, anemia, thrombocytopenia, nausea, and fatigue were evaluated in our analysis (Rashid et al., 2016; Mistry et al., 2018; Wong et al., 2018; Chen et al., 2021). The cost related to AEs was calculated by the cost of managing the AE per event by multiplying the incidence rate of each AE reported in the DESTINY-Breast03 trial. Drug doses and unit price are shown in Supplementary Table S3.

The utilities of the health states were obtained from the published literatures on advanced BC (Zhang and Long, 2019; Wu and Ma, 2020). We assigned utility values of 0.85 for all patients who either received T-DXd or T-DM1 in the PFS state and 0.52 for patients who moved to the PD state. The uncertainty surrounding the utility values was evaluated in the sensitivity analysis.

### Sensitivity analysis

To evaluate the robustness of the model and the variable uncertainty influence on the results, one-way sensitivity analysis was conducted with all parameters significant at 95% confidence intervals or within a range of 20% from their baseline values (Kohn et al., 2017; Wan et al., 2019). In the probabilistic sensitivity analysis (PSA), key model parameters were randomly sampled using the Monte Carlo simulations to run 10,000 replicated outcomes. We assigned recommended distributions according to the parameter types, with Gamma distribution representing the costs, and Beta distribution representing the probabilities, incidences of AEs and utility scores.

## Results

### Base-case results

Within a 10-year horizon, the life expectancy of patients with mBC receiving T-DXd was 0.63 LYs (7.56 months) longer than that of patients receiving T-DM1 based on the model. After applying quality-of-life adjustment and future discounting, T-DXd gained additional 0.73 QALYs compared with T-DM1. In the US, the use of T-DXd cost an additional $59,942 compared with T-DM1, resulting in an ICER of $82,112/QALY ($95,146/LY) and an INHB of 0.33 QALYs at a WTP threshold of $150,000/QALY. In China, the incremental cost of T-DXd versus T-DM1 was $222,680, with an ICER of $305,041/QALY ($353,460/LY) and an INHB of -5.18 QALYs at a WTP threshold of $37,653/QALY. Detailed results are showed in Table 3.

**TABLE 3:** Base-case results.

Strategy	Total cost ($)	Overall QALYs	Overall LYs	ICER ($)		INHB
				per LY	per QALY	
The US						
T-DXd	704,590	3.83	5.83	95,146	82,112	0.33
T-DM1	644,648	3.10	5.20	-	-	-
China						
T-DXd	533,251	3.83	5.83	353,460	305, 041	−5.18
T-DM1	310,571	3.10	5.20	-	-	-

ICER, incremental cost-effectiveness ratio; INHB, incremental net-health benefit; LY, life year; QALY, quality-adjusted life year; T-DM1, trastuzumab emtansine; T-DXd, trastuzumab deruxtecan.

### Sensitivity analysis

**FIGURE 2:**
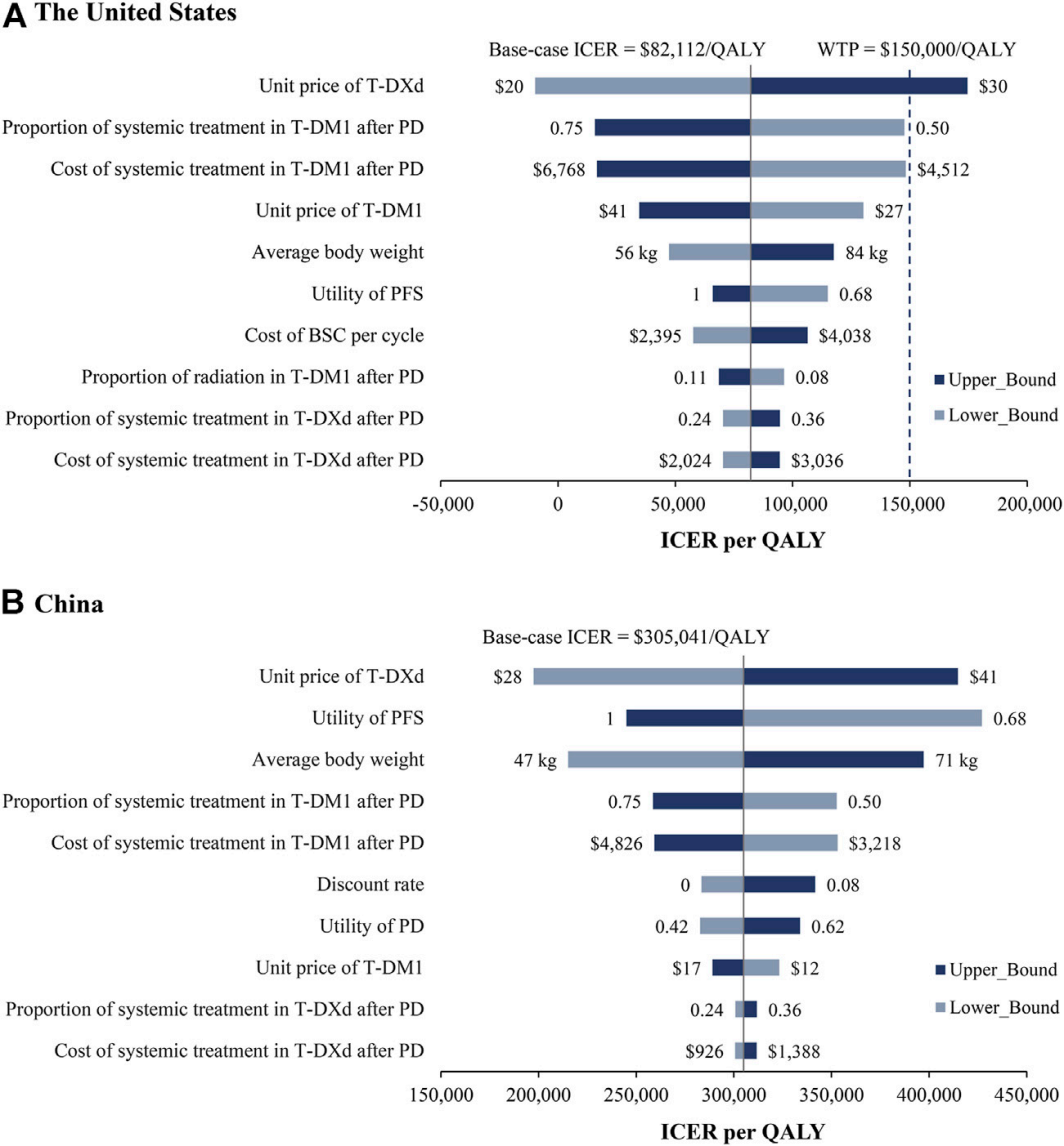
One-way sensitivity analyses. Tornado diagrams show the top 10 parameters that have the greatest impact on the results from the perspectives of the US **(A)** and China **(B)**. BSC, best support care; ICER, incremental cost-effectiveness ratio; PD, progression disease; PFS, progression-free survival; QALY, quality-adjusted life year.

Tornado diagrams are employed to present the results of the one-way sensitivity analysis (Figure 2). In the US, the unit price of T-DXd had the greatest impact on the ICER. When its lower boundary ($20/mg) was applied, the ICER of T-DXd vs. T-DM1 fell to $-9,716/QALY, which suggested that T-DXd gained more health benefits with less cost. When the upper boundary ($30/mg) was applied, the ICER increased to $174,541/QALY, which was greater than the threshold of $150,000/QALY. Other considerable influential parameters were the proportion and cost of patients receiving systematic treatment in the T-DM1 group after PD, the unit price of T-DM1, and the average body weight, which would not increase ICERs over the WTP threshold. Similar results were obtained from the perspective of China. The most sensitive parameters were the unit price of T-DXd, the utility of FPS, and the average body weight, which varied the ICERs ranged from $197,478/QALY to $414,833/QALY, $244,966/QALY to $427,051/QALY, and $215,166/QALY to $397,145/QALY, respectively. Regardless of the changes in the parameters, the ICERs were consistently higher than the WTP threshold of $37,653/QALY.

**FIGURE 3:**
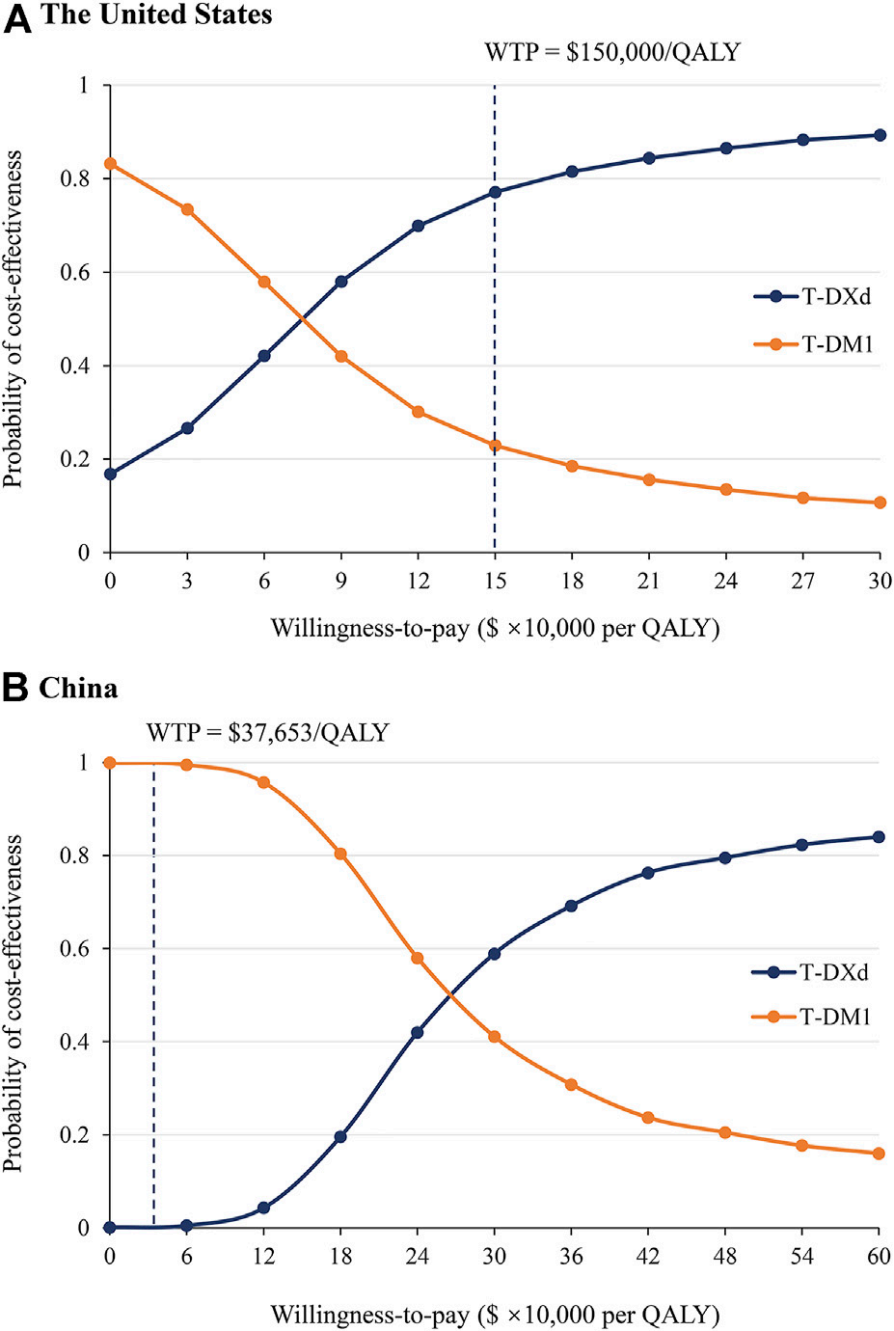
Probabilistic sensitivity analysis. Acceptable curves present the probability of T-DXd and T-DM1 being cost-effective at different WTP thresholds from the perspectives of the US **(A)** and China **(B)**. The dark dotted lines represent the thresholds used in the study. QALY, quality-adjusted life year; WTP, willingness-to-pay.

The results of PSA showed that compared with T-DM1, the probability of T-DXd being cost-effective is 77.5 and 0.1% when the threshold was equal to $150,000/QALY and $37,653/QALY in the US and China, respectively (Figure 3 and Supplementary Figure S3). When the price of T-DXd was reduced to 50 and 35% of its current price ($17.24/mg and $12.06/mg, respectively), there would be a more than 50 and 90% chance that T-DXd would be a cost-effective therapy in China.

**FIGURE 4:**
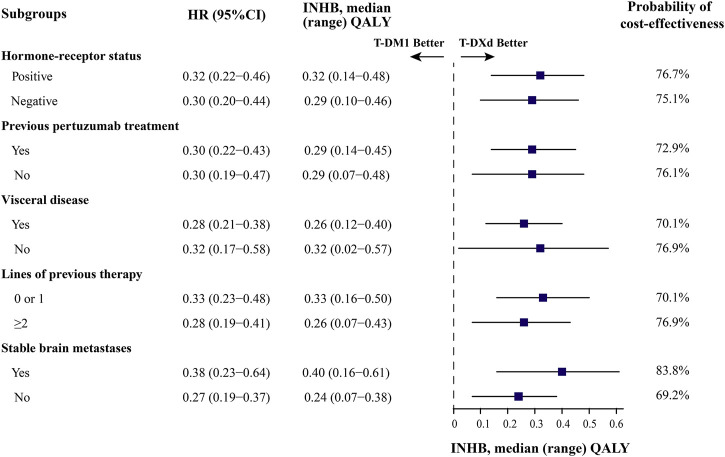
Subgroup analysis results of INHBs and probabilities of being cost-effective for progression-free survival in the US. The vertical dotted line represents the point of no effect (INHB = 0), the dark blue squares represent the median INHBs, and the horizontal lines represent the ranges of INHBs adjusted by the HRs. HR, hazard ratio; INHB, incremental net-health benefit; PFS, progression-free survival.

In subgroup analyses, T-DXd remained a cost-effective strategy from the perspective of the US. The ICER showed the greatest decrease in patients with stable brain metastases, with an ICER of $61,366/QALY and an INHB of 0.40 QALY, followed by patients treated with 0 or 1 line of previous therapy, patients with HR-positive disease, and patients without visceral disease (Figure 4 and Supplementary Table S4). Furthermore, T-DXd was bound up with positive INHBs, and the probability of T-DXd being cost-effective was greater than 70% in most subgroups at the WTP threshold of $150,000/QALY. In China, the lowest ICER among different subgroups was $289,078/QALY, which was well above the WTP threshold of $37,653/QALY. T-DXd was associated with negative INHBs in all subgroups with zero chance to be cost-effective (Supplementary Figure S4 and Supplementary Table S4).

## Discussion

The inspiring results of phase III DESTINY-Breast03 trial demonstrated a favorable benefit-risk profile for the novel ADC T-DXd with a significantly improved PFS over T-DM1. However, the high prices of new anticancer drugs impose economic burdens for both patients and the national medical healthcare systems, leading to a sharp increase in the consumption of health resources. Therefore, to make the best use of limited resources, it is important to make an economic evaluation of new therapies and expensive drugs.

Base on the current model, the T-DXd gained an additional 0.73 QALYs compared with T-DM1, providing ICERs of $82,112/QALY in the US and $305,041/QALY in China, respectively. At the WTP threshold of $150,000/QALY in the US, the results suggested that T-DXd was a cost-effective therapeutic approach. However, T-DXd was less cost-effective than T-DM1 in China given the current drug prices.

One-way sensitivity analysis suggested that the most relatively sensitive parameter driving this model outcome was the drug price of T-DXd in both the US and China. The PSA showed a 77.5% probability of T-DXd being cost-effective in the US, while T-DXd had nearly zero chance to be cost-effective in China. To achieve the same probability of cost-effectiveness, the appropriate unit price of T-DXd would be less than $15.18/mg (44% of current price), which indicated that the T-DXd was less acceptable in China than in the US. The potential reasons might be the following: the WTP threshold in the US was much higher than that in China, and the price gap between T-DXd and T-DM1 in China. At present, T-DM1 has been market in mainland China, but not T-DXd. The model’s drug price was referred to the Hongkong price. We noted that the price of T-DXd is more than three times that of T-DM1, and this gap leaded the total cost of T-DXd to be substantially higher than that of T-DM1, resulting in unfavorable results. Although T-DXd may not be cost-effective in China, this should not mean that patients should be treated with the less-effective therapies. The sensitivity analysis suggested that the economic outcome may become favorable as the price of T-DXd decreases. As part of the medical and health system program in the National 13th five-year plan, the Chinese government has launched a centralized drug procurement plan. Actions have been taken to reduce the price of anti-cancer drugs. And the prices of many anticancer drugs dropped sharply after negotiation and included in medical insurance after entering the Chinese market (State Council of China, 2020). Therefore, the actual variations in drug prices provide the possibility for T-DXd to be cost-effective after entering the procurement list in China.

Some previous studies have evaluated the cost-effectiveness of ADCs in BC. One recent publication analyzed the cost-effective of sacituzumab govitecan (SG), which has been approved by the US FDA for the treatment of metastatic triple-negative BC (Chen et al., 2021). Compared with single-agent chemotherapy, SG presented an ICER of $924,037/QALY in China and $494,479/QALY in the US, respectively, which indicated that SG was not cost-effective in both two countries. Another cost-effectiveness analysis evaluated data from the EMILIA and TH3RESA trials and found that the T-DM1 had ICERs of €167,236/QALY compared with lapatinib plus capecitabine, and €49,798/QALY compared with capecitabine (Squires et al., 2016). The results of PSA showed that T-DM1 was not cost-effective for treating HER2-positive advanced BC. Similar results with ADCs being not cost-effective were obtain in several study (Le et al., 2016; Diaby et al., 2017; Wang et al., 2020; Zhang et al., 2021). However, Guiliani et al. reported that T-DM1 was a cost-effective option for pretreated HER2-positive BC with an ICER of € 406 per month of OS gained (Giuliani and Bonetti, 2021). The difference result might mainly due to the different outcomes used.

Several strengths of present study deserve to be emphasized. First, to the best of our knowledge, this is the first cost-effectiveness analysis that directly compared T-DXd with T-DM1 in patients with HER2-positive mBC by incorporating the latest evidence. ADCs are emerging as promising therapeutic options for BC. Both T-DXd and T-DM1 have received approval from the FDA for metastasis HER2-positive BC, however, precious economic evaluation of T-DXd and T-DM1 is limited. Second, for the differences in medical environment and nation situation, we focused on comparing the cost-effectiveness of T-DXd and T-DM1 from the perspective of the US and the Chinese medical system. The results could provide useful information to the clinician, government, and the healthcare financial structures, to make decisions. Furthermore, the current analysis is helpful to inform the multilateral drug price negotiations with the availability of T-DXd in the Chinese market. Third, we conducted analysis to estimate the economic outcomes of five subgroups designated by the DESTINY-Breast03 trial. Subgroup based economic information may contribute to treatment decision-making.

Our analysis has some limitations need to be mentioned. First, as with many models, we used a log-logistic distribution to deduce the survival outcomes beyond the observational time of the DESTINY-Breast03 trial, which was an inevitable limitation. However, the model uncertainty regarding the long-term survival rates is small owing to the good fitness of the model. The long-term benefits of T-DXd remain an open question. The model can be verified based on long-term survival data when more mature data is available in the future. Second, patients in our model were assumed to have similar quality of life to those in previous studies because the DESTINY-Breast03 trial did not report the health utility values. Additionally, we assumed the utility values of Chinese patients were the same as those of Western patients. The utilities within a range of 20% were subjected to sensitivity analysis to confirm that this parameter would not change the results. Third, the cost of grade 1 or 2 AEs was not considered, which may underestimate the total cost of T-DXd. Fortunately, the results were not sensitive to the parameters associated with AEs. Fourth, since T-DXd has not been marketed in Chinese mainland, the model’s drug price was based on the Hongkong price. We estimate the cost-effectiveness of T-DXd by calculating 50 and 35% of the model price, which is expected to include the lowest price of T-DXd upon approval. The current study is required to be update as T-DXd is launched in China.

## Conclusion

In conclusion, T-DXd is a cost-effective therapy for patients with HER2-positive mBC compared to T-DM1 from the perspective of the US at a WTP threshold of $150,000/QALY. However, T-DXd is less cost-effective than T-DM1 in China at current drug price. A reduction in the price of T-DXd may be helpful to improve its cost-effectiveness.

## Data Availability

The original contributions presented in the study are included in the article/Supplementary Material, further inquiries can be directed to the corresponding author.

## References

[B1] Aguiar P. N. Jr. Haaland B. Park W. San Tan P. Del Giglio A. de Lima Lopes G. Jr (2018). Cost-effectiveness of osimertinib in the first-line treatment of patients with EGFR-mutated advanced non-small cell lung cancer. JAMA Oncol. 4, 1080–1084. 10.1001/jamaoncol.2018.1395 29852038 PMC6143050

[B2] Centers for Medicare and Medicaid Services (2022). Medicare Part B drug average sale price. Available at: https://www.cms.gov/medicare/medicare-part-b-drug-average-sales-price/2022-asp-drug-pricing-files (Accessed April 4, 2022).

[B3] Chen J. Han M. Liu A. Shi B. (2021). Economic evaluation of sacituzumab govitecan for the treatment of metastatic triple-negative breast cancer in China and the US. Front. Oncol. 11, 734594. 10.3389/fonc.2021.734594 34778047 PMC8581633

[B4] Chen X. Liang W. Wan N. Zhang L. Yang Y. Jiang J. (2019). Cost-effectiveness analysis of gemcitabine plus cisplatin versus fluorouracil plus cisplatin for first-line treatment of recurrent or metastatic nasopharyngeal carcinoma. Oral Oncol. 94, 80–85. 10.1016/j.oraloncology.2019.04.022 31178217

[B5] Cortes J. Kim S. B. Chung W. P. Im S. A. Park Y. H. Hegg R. (2022). Trastuzumab deruxtecan versus trastuzumab emtansine for breast cancer. N. Engl. J. Med. 386, 1143–1154. 10.1056/NEJMoa2115022 35320644

[B6] CSCO (2022). Chinnese society of clinical oncology. Available at: http://www.csco.org.cn/cn/index.aspx (Accessed April 15, 2022).

[B7] Deshmukh A. A. Shirvani S. M. Lal L. Swint J. M. Cantor S. B. Smith B. D. (2017). Cost-effectiveness analysis comparing conventional, hypofractionated, and intraoperative Radiotherapy for early-stage breast cancer. J. Natl. Cancer Inst. 109, 1–9. 10.1093/jnci/djx068 29059428

[B8] Diaby V. Adunlin G. Montero A. J. (2014). Survival modeling for the estimation of transition probabilities in model-based economic evaluations in the absence of individual patient data: A tutorial. Pharmacoeconomics 32, 101–108. 10.1007/s40273-013-0123-9 24338265

[B9] Diaby V. Ali A. A. Williams K. J. Ezendu K. Soto-Perez-de-Celis E. Chavarri-Guerra Y. (2017). Economic evaluation of sequencing strategies in HER2-positive metastatic breast cancer in Mexico: A contrast between public and private payer perspectives. Breast Cancer Res. Treat. 166, 951–963. 10.1007/s10549-017-4473-4 28840424

[B10] Ding H. Fang L. Xin W. Tong Y. Zhou Q. Huang P. (2017). Cost-effectiveness analysis of fulvestrant versus anastrozole as first-line treatment for hormone receptor-positive advanced breast cancer. Eur. J. Cancer Care 26, e12733. 10.1111/ecc.12733 28675545

[B11] DrugsHK (2022). Search cancer drugs. Available at: https://drugs-hk.squarespace.com/ (Accessed April 4, 2022).

[B12] FDA (2019). FDA approves fam-trastuzumab deruxtecan-nxki for unresectable or metastatic HER2-positive breast cancer. Available at: https://www.fda.gov/drugs (Accessed April 8, 2022).

[B13] Giuliani J. Bonetti A. (2021). The cost-effectiveness of trastuzumab emtansine (T-DM1) in HER2-positive metastatic breast cancer is supported by clinical evidence. Breast J. 27, 75–76. 10.1111/tbj.14024 32920921

[B14] Hoyle M. W. Henley W. (2011). Improved curve fits to summary survival data: Application to economic evaluation of health technologies. BMC Med. Res. Methodol. 11, 139. 10.1186/1471-2288-11-139 21985358 PMC3198983

[B15] Huntington S. F. von Keudell G. Davidoff A. J. Gross C. P. Prasad S. A. (2018). Cost-effectiveness analysis of brentuximab vedotin with chemotherapy in newly diagnosed stage III and IV hodgkin lymphoma. J. Clin. Oncol. 36, 3307–3314. 10.1200/JCO.18.00122 PMC6241679 30285558

[B16] Kohn C. G. Zeichner S. B. Chen Q. Montero A. J. Goldstein D. A. Flowers C. R. (2017). Cost-effectiveness of immune checkpoint inhibition in BRAF wild-type Advanced melanoma. J. Clin. Oncol. 35, 1194–1202. 10.1200/JCO.2016.69.6336 28221865 PMC5791832

[B17] Krop I. E. Kim S. B. Martin A. G. LoRusso P. M. Ferrero J. M. Badovinac-Crnjevic T. (2017). Trastuzumab emtansine versus treatment of physician's choice in patients with previously treated HER2-positive metastatic breast cancer (TH3RESA): Final overall survival results from a randomised open-label phase 3 trial. Lancet. Oncol. 18, 743–754. 10.1016/S1470-2045(17)30313-3 28526538

[B18] Le Q. A. Bae Y. H. Kang J. H. (2016). Cost-effectiveness analysis of trastuzumab emtansine (T-DM1) in human epidermal growth factor receptor 2 (HER2): Positive advanced breast cancer. Breast Cancer Res. Tr. 159, 565–573. 10.1007/s10549-016-3958-x 27572338

[B19] Mistry R. May J. R. Suri G. Young K. Brixner D. Oderda G. (2018). Cost-effectiveness of ribociclib plus letrozole versus palbociclib plus letrozole and letrozole monotherapy in the first-line treatment of postmenopausal women with hr+/HER2- advanced or metastatic breast cancer: A U.S. Payer perspective. J. Manag. Care Spec. Pharm. 24, 514–523. 10.18553/jmcp.2018.24.6.514 29799329 PMC10398120

[B20] Modi S. Saura C. Yamashita T. Park Y. H. Kim S. B. Tamura K. (2020). Trastuzumab deruxtecan in previously treated HER2-positive breast cancer. N. Engl. J. Med. 382, 610–621. 10.1056/NEJMoa1914510 31825192 PMC7458671

[B21] Murray C. J. Evans D. B. Acharya A. Baltussen R. M. (2000). Development of WHO guidelines on generalized cost-effectiveness analysis. Health Econ. 9, 235–251. 10.1002/(sici)1099-1050(200004)9:3<235::aid-hec502>3.0.co;2-o 10790702

[B22] Nakada T. Sugihara K. Jikoh T. Abe Y. Agatsuma T. (2019). The latest research and development into the antibody-drug conjugate, [fam-] trastuzumab deruxtecan (DS-8201a), for HER2 cancer therapy. Chem. Pharm. Bull. 67, 173–185. 10.1248/cpb.c18-00744 30827997

[B23] THE PEOPLE’S BANK OF CHINA (2022). Available at: http://www.pbc.gov.cn/rmyh/108976/109428/index.html (Accessed April 1, 2022).

[B25] NCCN (2022). NCCN guidelines, breast cancer. Available at: https://www.nccn.org/guidelines/category_1 (Accessed April 8, 2022).

[B26] Ogitani Y. Aida T. Hagihara K. Yamaguchi J. Ishii C. Harada N. (2016). DS-8201a, A novel HER2-targeting ADC with a novel DNA topoisomerase I inhibitor, demonstrates a promising antitumor efficacy with differentiation from T-DM1. Clin. Cancer Res. 22, 5097–5108. 10.1158/1078-0432.Ccr-15-2822 27026201

[B27] Posada D. Buckley T. R. (2004). Model selection and model averaging in phylogenetics: Advantages of akaike information criterion and bayesian approaches over likelihood ratio tests. Syst. Biol. 53, 793–808. 10.1080/10635150490522304 15545256

[B28] Rashid N. Koh H. A. Baca H. C. Lin K. J. Malecha S. E. Masaquel A. (2016). Economic burden related to chemotherapy-related adverse events in patients with metastatic breast cancer in an integrated health care system. Breast Cancer (Dove Med. Press) 8, 173–181. 10.2147/BCTT.S105618 27785099 PMC5063632

[B29] Sorensen S. V. Goh J. W. Pan F. Chen C. Yardley D. Martin M. (2012). Incidence-based cost-of-illness model for metastatic breast cancer in the United States. Int. J. Technol. Assess. Health Care 28, 12–21. 10.1017/S026646231100064X 22617734

[B30] Squires H. Stevenson M. Simpson E. Harvey R. Stevens J. (2016). Trastuzumab emtansine for treating HER2-positive, unresectable, locally advanced or metastatic breast cancer after treatment with trastuzumab and a taxane: An evidence review group perspective of a NICE single technology appraisal. Pharmacoeconomics 34, 673–680. 10.1007/s40273-016-0386-z 26892972

[B31] State Council of China (2020). China's centralized procurement leads to 50% drop in prices of over 100 drugs. Available at: http://english.www.gov.cn/statecouncil/ministries/202011/21/content_WS5fb86defc6d0f7257694042b.html (Accessed April 10, 2022).

[B32] Su D. Wu B. Shi L. (2021). Cost-effectiveness of atezolizumab plus bevacizumab vs sorafenib as first-line treatment of unresectable hepatocellular carcinoma. JAMA Netw. Open 4, e210037. 10.1001/jamanetworkopen.2021.0037 33625508 PMC7905498

[B33] Sung H. Ferlay J. Siegel R. L. Laversanne M. Soerjomataram I. Jemal A. (2021). Global cancer statistics 2020: GLOBOCAN estimates of incidence and mortality worldwide for 36 cancers in 185 countries. Ca. Cancer J. Clin. 71, 209–249. 10.3322/caac.21660 33538338

[B34] The State Council Information Office (2020). Report on nutrition and chronic disease status of Chinese Residents. Available at: http://www.gov.cn/xinwen/2020-12/24/content_5572983.htm (Accessed April 4, 2022).

[B35] US Department of Health and Human Services (2022). Medicare physician fee schedule (MFS). Available at: http://www.cms.gov/apps/physician-fee-schedule/overview.aspx (Accessed April 4, 2022).

[B36] Verma S. Miles D. Gianni L. Krop I. E. Welslau M. Baselga J. (2012). Trastuzumab emtansine for HER2-positive advanced breast cancer. N. Engl. J. Med. 367, 1783–1791. 10.1056/NEJMoa1209124 23020162 PMC5125250

[B37] Waks A. G. Winer E. P. (2019). Breast cancer treatment: A review. JAMA 321, 288–300. 10.1001/jama.2018.19323 30667505

[B38] Wan X. Zhang Y. Tan C. Zeng X. Peng L. (2019). First-line nivolumab plus ipilimumab vs sunitinib for metastatic renal cell carcinoma: A cost-effectiveness analysis. JAMA Oncol. 5, 491–496. 10.1001/jamaoncol.2018.7086 30789633 PMC6459127

[B39] Wang L. C. Kuo C. N. Ko Y. (2020). Cost-effectiveness analysis of trastuzumab emtansine (T-DM1) in treating HER-2 positive advanced breast cancer in Taiwan. Breast J. 26, 2099–2102. 10.1111/tbj.14053 32945063

[B40] Wong W. Yim Y. M. Kim A. Cloutier M. Gauthier-Loiselle M. Gagnon-Sanschagrin P. (2018). Assessment of costs associated with adverse events in patients with cancer. PLoS One 13, e0196007. 10.1371/journal.pone.0196007 29652926 PMC5898735

[B41] Wu B. Gu X. Zhang Q. (2018a). Cost-effectiveness of osimertinib for EGFR mutation-positive non-small cell lung cancer after progression following first-line EGFR TKI therapy. J. Thorac. Oncol. 13, 184–193. 10.1016/j.jtho.2017.10.012 29101057

[B42] Wu B. Ma F. (2020). Cost-effectiveness of adding atezolizumab to first-line chemotherapy in patients with advanced triple-negative breast cancer. Ther. Adv. Med. Oncol. 12, 1–12. 10.1177/1758835920916000 PMC7222249 32426048

[B43] Wu B. Zhang Q. Sun J. (2018b). Cost-effectiveness of nivolumab plus ipilimumab as first-line therapy in advanced renal-cell carcinoma. J. Immunother. Cancer 6, 124. 10.1186/s40425-018-0440-9 30458884 PMC6247499

[B44] Yaozh (2022). Yaozh. Available at: https://db.yaozh.com/ (Accessed April 13, 2022).

[B45] Zhang B. Long E. F. (2019). Cost-effectiveness analysis of palbociclib or ribociclib in the treatment of advanced hormone receptor-positive, HER2-negative breast cancer. Breast Cancer Res. Treat. 175, 775–779. 10.1007/s10549-019-05190-3 30847728

[B46] Zhang H. H. Zhang Y. D. Huang C. N. Wang J. F. (2021). Cost-effectiveness analysis of trastuzumab emtansine as second-line therapy for HER2-positive breast cancer in China. Clin. Drug Investig. 41, 569–577. 10.1007/s40261-021-01035-4 33876415

