# Supplementary material for: Cost-effectiveness analysis of trastuzumab deruxtecan versus trastuzumab emtansine for HER2-positive breast cancer
Source: Front Pharmacol. 2022 Sep 9;13:924126. doi: 10.3389/fphar.2022.924126 (PMC9500475; doi:10.3389/fphar.2022.924126)
Supplement: Supplementary file 1 [file DataSheet1.docx]

Supplementary Material

**Supplementary Table S1.** Patient baseline demographic and clinical characteristics.

**Supplementary Table S2.** Parametric survival distributions evaluated for progression-free survival and overall survival.

**Supplementary Table S3.** Drug doses and unit price in the United States and China.

**Supplementary Table S4.** Results for subgroup analyses and probabilities of cost-effectiveness by varying the HRs of the PFS.

**Supplementary Figure S1.** Markov model simulating the treatment arms in DESTINY-Breast03 Trial.

**Supplementary Figure S2.** Kaplan-Meier curve fitting and extrapolation

**Supplementary Figure S3.** Probability sensitivity analysis scatter plots.

**Supplementary Figure S4.** Subgroup analysis results of INHBs and probabilities of cost-effectiveness for progression-free survival in China.

**Supplementary References**

**Supplementary Table S1.** Patient baseline demographic and clinical characteristics.

| **Characteristics** | **Trastuzumab Deruxtecan**  **(N = 261), No. (%)** | **Trastuzumab Emtansine**  **(N = 263), No. (%)** |
| --- | --- | --- |
| Median age, years (range) | 54.3 (27.9-83.1) | 54.2 (20.2-83.0) |
| **ECOG performance status** | | |
| 0 | 154 (59) | 175 (66.5) |
| 1 | 106 (40.6) | 87 (33.1) |
| **Race** | | |
| White | 71 (27.2) | 72 (27.4) |
| Black | 10 (3.8) | 9 (3.4) |
| Asian | 152 (58.2) | 162 (61.6) |
| Others | 28 (10.8) | 20 (7.6) |
| **Geographic region** | | |
| Asia | 149 (57.1) | 160 (60.8) |
| North America | 17 (6.5) | 17 (6.5) |
| Europe | 54 (20.7) | 50 (19.0) |
| Rest of world | 41 (15.7) | 36 (13.7) |
| **HER2 status** | | |
| 3+ | 234 (89.7) | 232 (88.2) |
| 2+ with HER2 ISH­positive | 25 (9.6) | 30 (11.4) |
| 1+ | 1 (0.4) | 0 |
| **Hormone­receptor status** | | |
| Positive | 131 (50.2) | 134 (51.0) |
| Negative | 130 (49.8) | 129 (49.0) |
| **Stable brain metastases** | 62 (23.8) | 52 (19.8) |
| **Visceral disease** | 184 (70.5) | 185 (70.3) |
| **Previous treatment for mBC** | 240 (92.0) | 234 (89.0) |
| **Median number of lines (range)** | 1 (0–16) | 2 (0–14) |
| **Previous cancer therapy** |  |  |
| Trastuzumab | 260 (99.6) | 262 (99.6) |
| Pertuzumab | 162 (62.1) | 158 (60.1) |

The baseline demographic and clinical characteristics of the hypothetical patients in the model were derived from the relevant data of the DESTINY­Breast03 Clinical Trial (1). mBC, metastatic breast cancer.

**Supplementary Table S2.** Parametric survival distributions evaluated for progression-free survival and overall survival.

| **Regimen** | **Model** | **AIC** | **BIC** |
| --- | --- | --- | --- |
| **Progression-free survival** | | | |
| **T-DXd** | Exponential | -193.1654 | -189.6901 |
|  | Weibull | -204.3638 | -199.1508 |
|  | Log-Logistic* | -234.078 | -228.865 |
|  | Log-Normal | -224.9509 | -219.7379 |
|  | Gompertz | -212.5075 | -207.2944 |
| **T-DM1** | Exponential | -87.44075 | -83.78346 |
|  | Weibull | -140.7788 | -135.2929 |
|  | Log-Logistic* | -170.3405 | -164.8546 |
|  | Log-Normal | -169.3175 | -163.8316 |
|  | Gompertz | -70.6308 | -65.14488 |
| **Overall survival** | | | |
| **T-DXd** | Exponential | -222.5904 | -218.848 |
|  | Weibull | -223.9301 | -218.3165 |
|  | Log-Logistic* | -227.0345 | -221.4209 |
|  | Log-Normal | -234.0997 | -228.4861 |
|  | Gompertz | -228.9333 | -223.3196 |
| **T-DM1** | Exponential | -201.5341 | -197.9208 |
|  | Weibull | -201.5411 | -196.1211 |
|  | Log-Logistic* | -214.148 | -208.728 |
|  | Log-Normal | -206.5756 | -201.1556 |
|  | Gompertz | -193.1102 | -187.6902 |

AIC, Akaike's information criterion; BIC, Bayesian's information criterion; T-DXd, trastuzumab deruxtecan; T-DM1, trastuzumab emtansine.

*the model was used to fit the Kaplan-Meier.

**Supplementary Table S3.** Drug doses and unit price in the United States and China.

| **Drug** | **Dose** | **Unit price ($)** | **Cost for one cycle ($/3 weeks)** | **Reference** |
| --- | --- | --- | --- | --- |
| **China** |  |  |  |  |
| **Progression free disease** |  |  |  |  |
| Trastuzumab deruxtecan | 5.4 mg/kg × 59kg, intravenously on day 1 of 21-day cycle | 3,447.18/100mg | 10,982.71 | (1-3) |
| Trastuzumab emtansine | 3.6 mg/kg × 59kg, intravenously on day 1 of 21-day cycle | 1,448.61/100mg | 3,077 | (1,3,4) |
| **Progression disease** |  |  |  |  |
| **Systemic therapy** |  |  |  |  |
| Trastuzumab | 8mg/kg followed by 6mg/kg × 59kg, intravenously on day 1 of 21-day cycle | 866.02/440mg | 696.75 | (1,3,4) |
| Trastuzumab deruxtecan | 5.4 mg/kg × 59kg, intravenously on days 1 of 21-day cycle | 3,447.18/100mg | 10,982.71 | (1-3) |
| Trastuzumab emtansine | 3.6 mg/kg × 59kg, intravenously on day 1 of 21-day cycle | 1,448.61/100m | 3,077 | (1,3,4) |
| Pertuzumab | 840mg followed by 420mg, intravenously on day 1 of 21-day cycle | 780.20/420mg | 780.20 | (1,3,4) |
| Taxane | 175 mg/m^2^ × 1.61 m^2^ on day 1 of 21-day cycle | 28.03/60mg | 131.62 | (1,3,4) |
| Other anti-HER2 |  |  |  |  |
| Anti-HER2 TKI | lapatinib:1250mg, orally once daily of 21-day cycle | 10.50/0.25g | 1,102.5 | (1,3,4) |
| Anti-HER2 antibody or  ADC | sacituzumab govitecan:  10 mg/kg × 59kg, intravenously on days 1 and 8 of 21-day cycle | 5,363/180mg | 35,157.44 | (1-3) |
| **Radiation *** | - | - | 6,298 | Estimated |
| **Surgery *** | - | - | 2,362 | Estimated |
| **the United States** |  |  |  |  |
| **Progression free disease** |  |  |  |  |
| Trastuzumab Deruxtecan | 5.4 mg/kg × 70kg, intravenously on day 1 of 21-day cycle | 24.617/mg | 9,305.23 | (1,5) |
| Trastuzumab Emtansine | 3.6 mg/kg × 70kg, intravenously on day 1 of 21-day cycle | 34.139/mg | 8,603.03 | (1,5) |
| **Progression disease** |  |  |  |  |
| **Systemic therapy** |  |  |  |  |
| Trastuzumab | 8mg/kg followed by 6mg/kg × 70kg, intravenously on day 1 of 21-day cycle | 70.957/10mg | 2,980.19 | (1,5) |
| Trastuzumab deruxtecan | 5.4 mg/kg × 70kg, intravenously on day 1 of 21-day cycle | 24.617/mg | 9,305.23 | (1,5) |
| Trastuzumab emtansine | 3.6 mg/kg × 70kg, intravenously on day 1 of 21-day cycle | 34.139/mg | 8,603.03 | (1,5) |
| Pertuzumab | 840mg followed by 420mg, intravenously on days1 of 21-day cycle | 13.556/mg | 5,693.52 | (1,5) |
| Taxane | 175 mg/m^2^ × 1.79 m^2^ day 1; every 21 days | 0.128/mg | 40.10 | (1,5) |
| Other anti-HER2 |  |  |  | (1,5) |
| Anti-HER2 TKI | lapatinib: 1250mg, orally once daily of 21-day cycle | 51.19/250mg | 5,374.95 | (1,5) |
| Anti-HER2 antibody/  ADC | sacituzumab govitecan: 10 mg/kg × 70kg, intravenously on days 1 and 8 of 21-day cycle | 31.258/2.5mg | 19,379.96 | (1,5) |
| **Radiation** | - | - | 7,814 | (1,6,7) |
| **Surgery** | - | - | 2,580 | (1,6,7) |

***** The costs of radiation and surgery were estimated based on the price of West China Hospital Sichuan University, 2022.

**Supplementary Table S4.** Results for subgroup analyses and probabilities of cost-effectiveness by varying the HRs of the PFS.

| **Subgroups** | **HR (95% CI)** | **ICER per QALY** | **Cost-effectiveness probability (%)** | **INHB** |
| --- | --- | --- | --- | --- |
|  |  |  |  |  |
| **The US perspective** | | | | |
| **Hormone-receptor status** | | | | |
| Positive | 0.32 (0.22–0.46) | 84,323 (30,677-125,963) | 76.7 | 0.32 (0.14-0.48) |
| Negative | 0.30 (0.20–0.44) | 92,857 (38,050-134,176) | 75.1 | 0.29 (0.10-0.46) |
| **Previous pertuzumab treatment** | | | | |
| Yes | 0.30 (0.22–0.43) | 92,857 (41,741-125,963) | 72.9 | 0.29 (0.14-0.45) |
| No | 0.30 (0.19–0.47) | 92,857 (26,983-138,553) | 76.1 | 0.29 (0.07-0.48) |
| **Visceral disease** | | | | |
| Yes | 0.28 (0.21–0.38) | 100,430 (61,366-129,986) | 70.1 | 0.26 (0.12-0.40) |
| No | 0.32 (0.17–0.58) | 84,323 (-14,562-146,457) | 76.9 | 0.32 (0.02-0.57) |
| **Lines of previous therapy** | | | | |
| 0 or 1 | 0.33 (0.23–0.48) | 81,299 (22,883-122,090) | 70.1 | 0.33 (0.16-0.50) |
| ≥ 2 | 0.28 (0.19–0.41) | 100,430 (49,928-138,553) | 76.9 | 0.26 (0.07-0.43) |
| **Stable brain metastases** | | | | |
| Yes | 0.38 (0.23–0.64) | 61,366 (-36,802-122,090) | 83.8 | 0.40 (0.16-0.61) |
| No | 0.27 (0.19–0.37) | 105,111 (65,247-138,553) | 69.2 | 0.24 (0.07-0.38) |
| **China perspective** | | | | |
| **Hormone-receptor status** | | | | |
| Positive | 0.32 (0.22–0.46) | 306,031 (264,510-342,140) | 0 | -5.27 (-7.12 to -3.61) |
| Negative | 0.30 (0.20–0.44) | 314,538 (268,858-348,350) | 0 | -5.59 (-7.59 to -3.81) |
| **Previous pertuzumab treatment** | | | | |
| Yes | 0.30 (0.22–0.43) | 314,538 (271,205 -342,140) | 0 | -5.59 (-7.12 to -3.91) |
| No | 0.30 (0.19–0.47) | 314,538 (262,503 -352,076) | 0 | -5.59 (-7.85 to -3.52) |
| **Visceral disease** | | | | |
| Yes | 0.28 (0.21–0.38) | 319,808 (289,078 -345,044) | 0 | -5.92 (-7.35 to -4.47) |
| No | 0.32 (0.17–0.58) | 306,031 (228,258 -357,244) | 0 | -5.27 (-8.40 to -2.63) |
| **Lines of previous therapy** | | | | |
| 0 or 1 | 0.33 (0.23–0.48) | 306,265 (256,188 -339,619) | 0 | -5.14 (-6.90 to -3.42) |
| ≥ 2 | 0.28 (0.19–0.41) | 319,808 (280,581 -352,076) | 0 | -5.92 (-7.85 to -4.13) |
| **Stable brain metastases** | | | | |
| Yes | 0.38 (0.23–0.64) | 289,078 (209,351 -339,619) | 0.2 | -4.47 (-6.90 to -2.23) |
| No | 0.27 (0.19–0.37) | 324,836 (292,196 -352,076) | 0 | -6.10 (-7.85 to -4.60) |

HR, hazard ratio; ICER, incremental cost-effectiveness ratio; INHB, incremental net-health benefits; PFS, progression-free survival; QALY, quality-adjusted life year.

**Supplementary Figure S1.** Markov model simulating the treatment arms in DESTINY-Breast03 Trial.


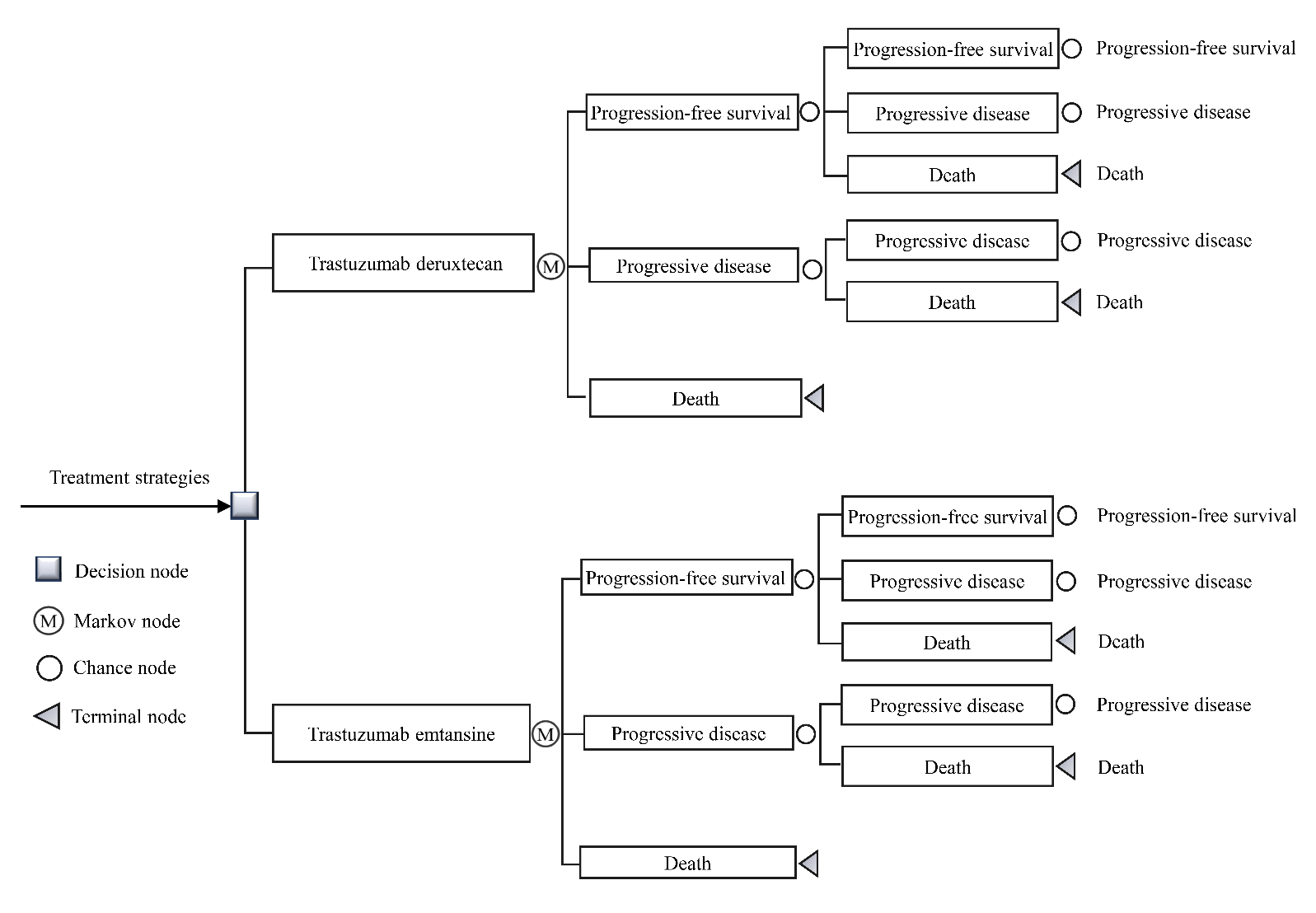


Markov model was used to simulate the course of disease for patients with metastatic breast cancer in the DESTINY-Breast03 trial. All patients entered the model in progression-free survival state and were randomly treated with trastuzumab deruxtecan or trastuzumab emtansine, with or without adverse events. All patients could continue treatment in response to therapy, or experience disease progression and receive the post-study anticancer treatment and best supportive care until death. It was assumed that the patients could not go back to previous health states.

**Supplementary Figure S2.** Kaplan-Meier curve fitting and extrapolation.

**A. Progression-free survival**

**
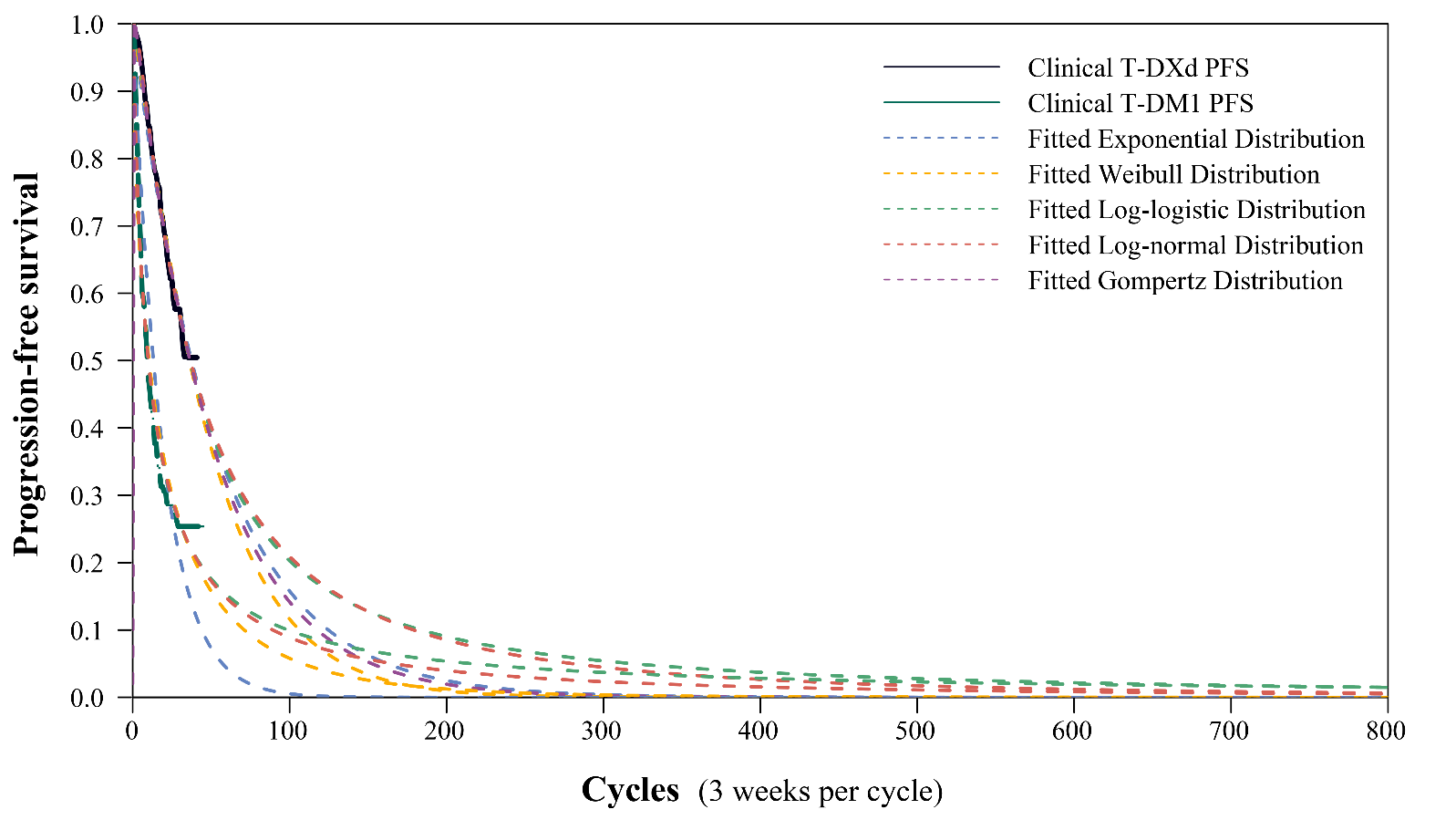
**

**B. Overall survival**

**
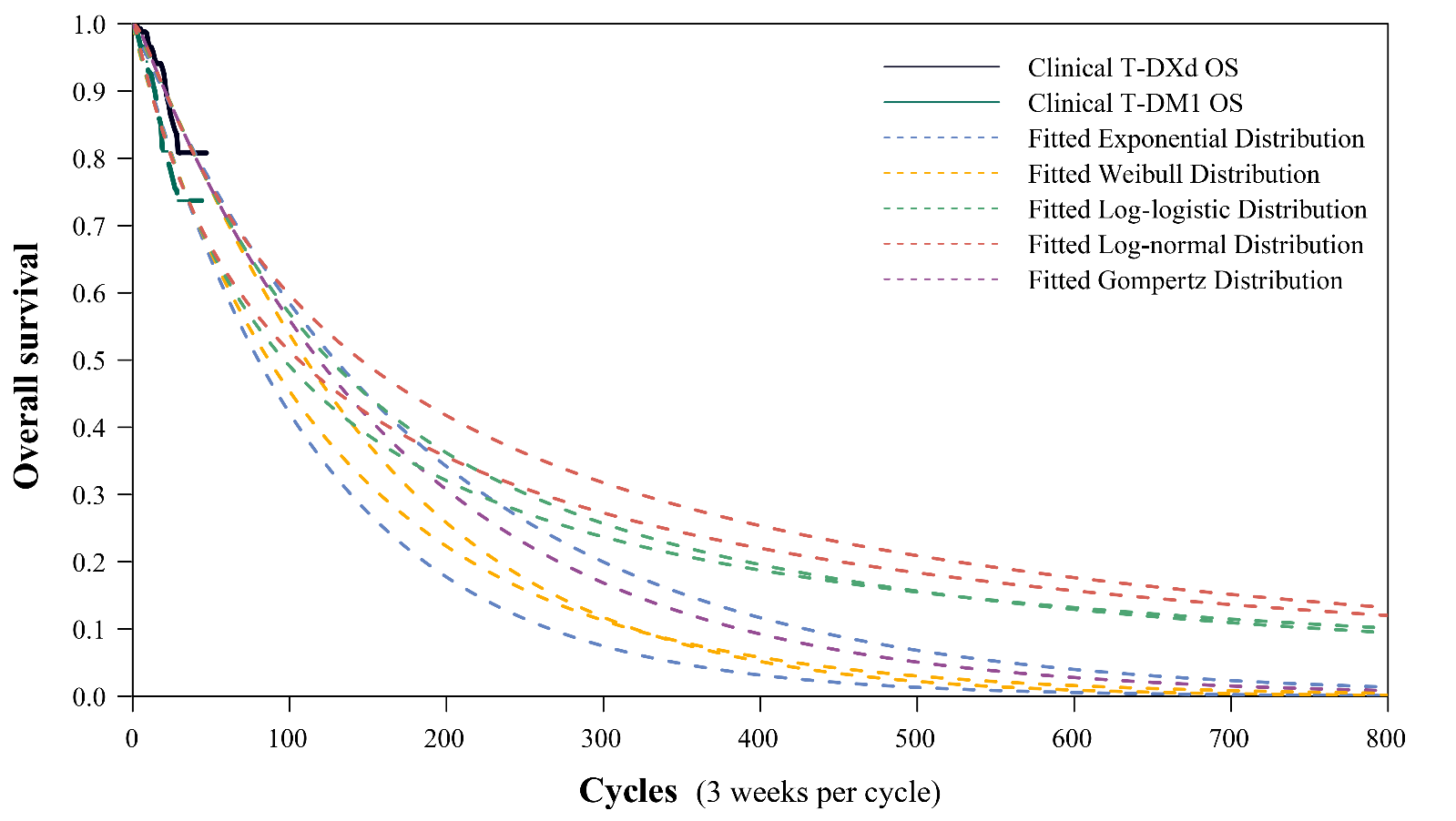
**

The solid lines represented original Kaplan-Meier curves from the DESTINY-Breast03 trial. The dotted line represented the validation of our model of treatment strategies for breast cancer.

PFS, progression-free survival; OS, overall survival; T-DXd, trastuzumab deruxtecan; T-DM1, trastuzumab emtansine.

**Supplementary Figure S3.** Probability sensitivity analysis scatter plots.


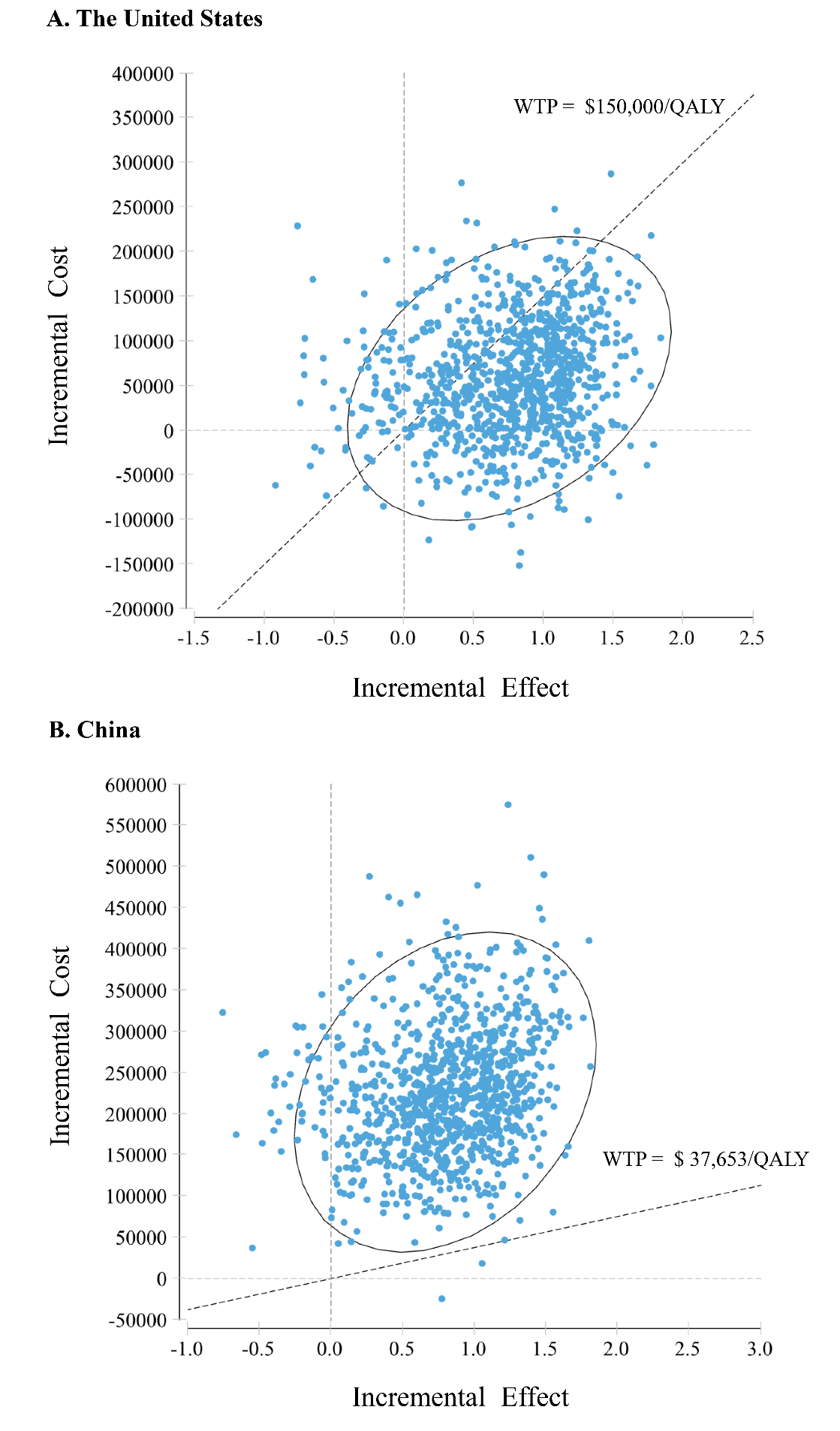


Each point in the diagram represents a simulation result of 10,000 Monte Carlo simulation. Ellipses represent 95% confidence intervals. Dotted dark line represent the willingness-to-pay threshold and points under the dark dotted line are considered cost-effective.

**Supplementary Figure S4.** Subgroup analysis results of INHBs and probabilities of cost-effectiveness for progression-free survival in China.


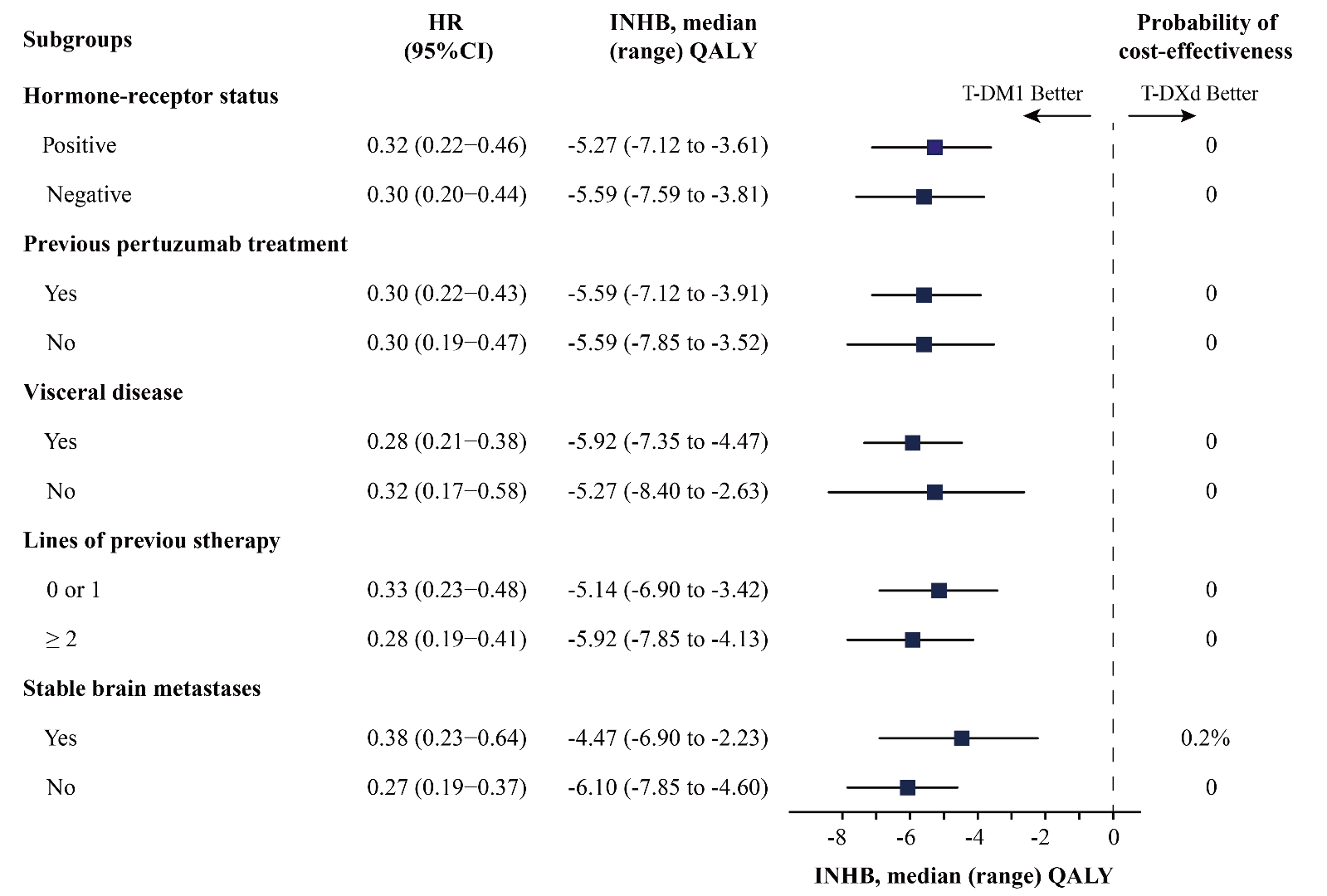


The vertical dotted line indicates the point of no effect (INHB = 0), the dark blue squares indicate the median INHB, and the dark bars indicate the ranges of INHBs adjusted by the HRs.

**Supplementary References:**

1. Cortes J, Kim SB, Chung WP, Im SA, Park YH, Hegg R, et al. Trastuzumab Deruxtecan versus Trastuzumab Emtansine for Breast Cancer. *N Engl J Med*. (2022) 386:1143-1154. doi: 10.1056/NEJMoa2115022.

2. DrugsHK. Available at: <https://drugs-hk.squarespace.com/> (Accessed 4 April 2022).

3. Report on Nutrition and Chronic Disease Status of Chinese Residents (2020). Available at: <http://www.gov.cn/xinwen/2020-12/24/content_5572983.htm> (Accessed 4 April 2022).

4. Yaozh. Available at: <https://db.yaozh.com/> (Accessed 13 April 2022).

5. Centers for Medicare and Medicaid Services: Medicare Part B Drug Average Sale Price. Available at: <https://www.cms.gov/medicare/medicare-part-b-drug-average-sales-price/2022-asp-drug-pricing-files> (Accessed 4 April 2022).

6. US Department of Health and Human Services. Medicare physician fee schedule (MFS) Available at: <http://www.cms.gov/apps/physician-fee-schedule/overview.aspx>. (Accessed 4 April 2022).

7. Deshmukh AA, Shirvani SM, Lal L, Swint JM, Cantor SB, Smith BD, et al. Cost-effectiveness Analysis Comparing Conventional, Hypofractionated, and Intraoperative Radiotherapy for Early-Stage Breast Cancer. *J Natl Cancer Inst*. (2017) 109. doi: 10.1093/jnci/djx068.
